# The Impact of Medical Insurance on Household Stock Market Participation: Evidence From China Household Finance Survey

**DOI:** 10.3389/fpubh.2021.710896

**Published:** 2021-07-26

**Authors:** Gui Fen Shi, Mei Li, Tao-Tao Shen, Yue Ma

**Affiliations:** School of Economics and Management, Northeast Normal University, Changchun, China

**Keywords:** medical insurance, household stock market participation, China household finance survey, preventive savings effect, financial development

## Abstract

This paper explores the impact of medical insurance on the possibility of household participation in the stock market and the portfolio share of equity, applying Probit, and Tobit models with the data from China Household Finance Survey (CHFS). The empirical results highlight that participating in medical insurance can significantly increase the possibility of households participating in the stock market and the portfolio share of equity, and have passed the robustness tests, including propensity score matching (PSM), altering estimation methods, replacing explained variables, and eliminating samples. Besides, heterogeneity analysis shows that the impact of medical insurance on household stock market participation is more significant in eastern region, urban areas, and households with higher income level. Further mechanism analysis implies that household participation in medical insurance mainly affects their stock market participation through preventive savings effect. It is necessary to improve the medical insurance system and encourage household participation in stock market so as to further promote financial development in China.

## Introduction

This paper aims to explore whether the possibility of household participation in the stock market responds to medical insurance, and how the participation in medical insurance affects the portfolio share of equity. It reveals the mystery of limited participation in the stock market from a new perspective, thereby providing new ideas for optimizing the allocation of household financial assets. With the development of Chinese financial market, the number of listed companies in China has increased from 1,088 in 2000 to 3,777 in 2019, and the amount of stock financing has also risen from 210.3 billion yuan in 2000 to 1,253.9 billion yuan in 2019. However, the proportion of household financial assets of Chinese urban households is only 11.8% in 2017, much lower than in developed countries such as the United States (42.6%) and Japan (61.1%)^1^. According to China Household Finance Survey (CHFS) statistics in 2015, the participation rate of Chinese household stock market accounts for only 9.3%, and the proportion of equity asset is 3.0%. Besides, China has a distinct dual economic structure and unbalanced regional economic development; thus, household stock market participation has shown limited and unbalanced characteristics (1). These two features have adverse effect on the preservation and appreciation of household wealth, as well as affect the diversified development of Chinese financial market.

The main factors that limit the participation of household stock markets can be explained in five aspects. First of all, high transaction cost and complex tax issues in stock market result in low investment motivation (2). Second, social factors such as risk attitudes, social capital, social network, and social interaction should be considered by households participating in the stock market (3–5). Then, demographic and household characteristic variables such as age, gender, marital status, population size, demographic structure, education level, income level, and wealth status have gradually been incorporated into the influence factors of the household stock market participation with the popularization of microsurvey data (6–8). Besides, financial knowledge and financial literacy, which affect the ability of decision-makers to identify and analyze financial asset information, are also important factors that affect household asset allocation. Residents with a higher level of financial knowledge not only have a higher participation rate, but also have a more diversified allocation types (9–11). Finally, as the existence of background risk such as labor income risks, health and life risks, the increase of household precautionary savings has a crowding-out effect on financial market participation (12, 13). With the improvement of living standards and insurance awareness, residents put more emphasis on health and life risks, and their demand for life security has increased. Scholars have begun to focus on the relationship between social security system and household risky asset investment. According to recent studies, the social security system can increase the breadth and depth of risky asset investment (14, 15). But related studies on the relationship between medical insurance and the participation in stock market are few.

In summary, this paper may have the following marginal contributions. First, most studies on the influence factors of household stock market participation mainly focus on the analysis of transaction costs, social factors, household, and demographic variables. We attempt to study the impact on household participation in stock market from the perspective of medical insurance, and test the influence mechanism at the same time. Second, based on the imbalance of Chinese regional economic development and the distinctive features of the urban–rural dual economic structure, we also analyze the heterogeneity from different perspectives of regions, urban–rural, and household income level. Third, we use the propensity score matching (PSM) method to deal with the possible endogeneity, and a variety of robust methods are used to verify the accuracy and reliability of the empirical results.

The rest of the paper is structured as follows. Section Literature Review and Research Hypothesis reviews the existing literature and proposes research hypothesis. Section Data, Variables and Empirical Models presents data, variables, and empirical models. Section Empirical Results and Analysis presents empirical results and corresponding analysis, and Section Conclusions and Policy Recommendations offers research conclusions and policy recommendations.

## Literature Review and Research Hypothesis

Classical asset portfolio theory believes that household makes corresponding investment plans according to their estimates of costs and benefits and their own risk-bearing capacity, so as to maximize household welfare (16–18). It is generally believed that household should hold a certain percentage of risky financial assets as long as there is a positive risk premium (19). However, Chinese households still face the problem of limited participation in risky assets, especially the stock market. According to an authoritative survey, Chinese household stock market participation rate in 2014 is only 6.5% (20). To explore the reasons for the limited participation of household financial assets in China, background risks such as labor income risks and health and life risks cannot be ignored. Health and life risks are important factors faced by Chinese households, and medical and health care expenditures account for a large proportion of the total household income and expenditures. According to statistics in 2018, the medical expenses for inpatients in general hospitals in China is 10,124.6 yuan per capita, while the urban disposable income per capita is 39,250.8 yuan, and the rural disposable income per capita is 14,617.0 yuan in the corresponding year^2^. Medical expenses account for about 30% of urban and 70% of rural disposable income per capita. It shows that the existence of diseases and health risks may cause households to fall into economic difficulties at any time, especially for rural households, which may further affect the household participation in the stock market.

Medical insurance, as an important part of the social security system, provides a health and life safety net for the insured households, reduces the uncertainty of future expenditures of residents, and has an important impact on the participation of Chinese household in the stock market. China has set up three major medical insurance systems from 1998 to 2007^3^. The establishment of the medical insurance system effectively alleviates the burden of the actual medical expenses and virtually increases the household wealth (21). The precautionary savings theory shows that the reduction of the risk of future large expenditures will prompt the household to reduce precautionary savings (22). According to Keynes's theory of money demand, as households invest less in low-risk investments such as bank deposits, they will put more assets into risky financial markets (23). It can be drawn that participating in medical insurance has an impact on household investment behavior by reducing precautionary savings, which is so-called the preventive savings effect of medical insurance.

The preventive savings effect of medical insurance has an important impact on household consumption, investment, and other behaviors. For household consumption, fewer household savings mean higher household consumption under the condition of certain income. Gan et al. (24) find that Chinese urban household consume more than 200 billion yuan in additional consumption due to medical insurance of urban residents, while new rural cooperative medical insurance and medical insurance for urban employees drive two to four times the number of rural and urban household consumption, respectively. Zang et al. (25) prove that annual nonmedical consumption expenditure of urban insured households is 13.0% higher than that of noninsured households. Bai and Wu (26) hold that the new rural cooperative medical insurance increases the consumption of insured household by 5.5%. For household investment, according to Kimball's risk aversion theory, households participating in medical insurance can obtain a certain degree of protection in disease and health; therefore, they will readjust the matching of returns and risks, and increase the investment of higher-risk assets accordingly (27). Wu and Zhou (28) propose that medical insurance can significantly affect the household financial asset allocation and increase the possibility of risky asset participation and the proportion of investment (29). Yi et al. (30) focus on the relationship between commercial medical insurance and household financial asset allocation, and find that commercial medical insurance can significantly increase the participation rate and the share of risky financial assets. It shows that the preventive savings effect can not only increase the probability of households participating in the stock market, but increase the depth of household participation in the stock market as well.

Based on the above analysis, this paper proposes the following research hypotheses.

H1: Participating in medical insurance can significantly increase the possibility of household participation in the stock market and the proportion of equity asset.
H2: Medical insurance mainly affects household stock market participation through preventive savings effect.

Chinese dual economic structure restricts the development of the rural economy, and there is a big difference in disposable income per capita between urban and rural areas. Chinese urban disposable income per capita is 31,195 yuan, while that in rural areas is 11,422 yuan in 2015^4^. The disparity of economic development between urban and rural areas will inevitably lead to great gap in the level of financial development and financial basic services. In addition, the sharp differences in education and financial knowledge of urban and rural residents are also important factors in determining whether they can obtain financial information in timely and effective manners. According to CHFS statistics in 2015, the participation rate and proportion of urban households in stock are 12.6 and 4.1%, respectively, while those of rural households are only 0.5 and 0.2% (Table 1). Furthermore, the participation rate and the proportion of stock assets of households in the eastern region are 13.5 and 4.3%, respectively, while those in the central region are 5.2 and 1.7%, and those in the western region are 5.4 and 1.8% (Table 2). Therefore, there are great differences in stock market participation between urban–rural areas and regions. Compared with underdeveloped areas and rural areas, developed areas and urban household have generally higher participation probability and investment shares in the stock market.

**Table 1:** Household financial asset allocation (overall and urban and rural).

**Asset class**	**Overall**			**Urban**			**Rural**		
	**Part rate (%)**	**Scale (¥)**	**Pro (%)**	**Part rate (%)**	**Scale (¥)**	**Pro (%)**	**Part rate (%)**	**Scale (¥)**	**Pro (%)**
Cash	98.14	6,896.68	38.39	98.23	7,393.14	32.25	97.88	5,612.74	54.28
Current deposit	77.05	30,356.10	37.13	81.96	36,270.38	38.54	64.36	15,060.86	33.49
Time deposit	23.76	23,120.77	14.66	26.76	29,014.82	16.01	16.02	7,877.84	11.16
Stock	9.26	15,415.27	2.99	12.62	20,972.33	4.08	0.54	1,043.83	0.17
Fund	4.04	3,300.14	1.11	5.44	4,495.45	1.50	0.42	208.89	0.10
Bond	0.64	742.07	0.17	0.83	927.29	0.23	0.15	263.04	0.02
Finance products	11.91	11,898.43	3.47	15.72	15,902.59	4.59	2.06	1,543.04	0.58
Derivative	0.08	365.00	0.01	0.11	506.14	0.02	0.00	0.00	0.00

*Part rate represents the participation rate, Scale is the holding scale, and Pro represents the proportion of asset allocation*.

**Table 2:** Household financial asset allocation (eastern, central, and western regions).

**Asset class**	**Eastern**			**Central**			**Western**		
	**Part rate (%)**	**Scale (¥)**	**Pro (%)**	**Part rate (%)**	**Scale (¥)**	**Pro (%)**	**Part rate (%)**	**Scale (¥)**	**Pro (%)**
Cash	98.01	8,919.16	33.79	98.54	4,962.05	46.26	97.91	5,007.64	38.25
Current deposit	79.15	40,498.18	35.37	69.68	19,552.62	36.18	81.97	22,292.63	42.12
Time deposit	28.74	31,787.81	17.14	18.01	14,126.90	11.99	20.38	15,925.16	12.72
Stock	13.47	25,819.98	4.30	5.15	6,327.93	1.73	5.42	4,590.25	1.78
Fund	5.58	5,466.39	1.48	2.16	1,296.54	0.58	3.10	1,189.18	0.98
Bond	0.92	1,212.78	0.27	0.44	398.41	0.07	0.30	166.06	0.10
Finance products	16.35	19,720.20	4.84	6.58	4,784.00	1.75	9.16	4,122.65	2.72
Derivative	0.10	228.81	0.01	0.06	587.72	0.01	0.04	373.97	0.03

*Part rate represents the participation rate, Scale is the holding scale, and Pro represents the proportion of asset allocation*.

Based on the above analysis, this paper proposes the following research hypothesis.

H3: The positive promotion of Chinese medical insurance on household stock market participation is more significant in eastern region, urban households, and households with higher income.

## Data, Variables, and Empirical Models

### Data

The data are from CHFS organized by the Survey and Research Center for China Household Finance of Southwestern University of Finance and Economics in 2015. The survey covers 363 counties and 1,439 village committees, involving 37,289 households and 125,248 individuals. In this paper, the age of the householder in the samples is 16 years old and over, and samples with missing values for the variables are removed to obtain samples containing 11,863 households. In addition, the data of the GDP per capita of the province representing the level of regional economic development are from the National Bureau of Statistics.

### Descriptive Statistics

The explained variables are the possibility of household participation in the stock market and the proportion of equity asset. In the robustness test, we use the possibility of household holding risky financial assets, proportion of risky financial assets, and types of risky financial assets. The key explanatory variable is based on whether the householder participates in medical insurance (31)^5^. Drawing on existing literature (32, 33), three types of characteristic variables are introduced as control variables. The first is personal characteristic variables of householders, including gender, marital status, education, risk preference, financial knowledge, financial attention, and state of health. The second is household characteristic variables, including the total household population, the proportion of the elderly, the income per capita of the household, wealth, whether the household owns a house, social interaction, and degree of trust. The third is regional characteristic variables, mainly considering the GDP per capita of the province. The descriptive statistics of related variables are shown in Table 3.

**Table 3:** Variable description and descriptive statistics.

**Variables**	**Definition**	**Mean**	**Standard deviation**	**Minimum**	**Maximum**
**Explained variables**					
Stock_if	Yes (1), no (0)	0.09	0.29	0.00	1.00
Stock_rate	Stock ratio (%)	2.93	11.77	0.00	100
**Core explanatory variables**					
Medical_if	Yes (1), no (0)	0.94	0.24	0.00	1.00
**Personal characteristic variables of householder**					
Male	Male (1), Female (0)	0.54	0.50	0.00	1.00
Age	Age of householder	50.22	14.17	17.00	91.00
Age^2^/100	Age^2^/100	27.23	14.52	2.89	82.81
Married	Yes (1), no (0)	0.88	0.33	0.00	1.00
Edu	Non education (0), primary school (6), junior high school (9), senior high school (12), technical secondary school (13), junior college (15), undergraduate college (16), graduate student (19), and doctoral student (22)	9.81	4.05	0.00	22.00
Attitude	Risk preference (2), risk neutral (1), and risk aversion (0)	0.40	0.67	0.00	2.00
Know	The ability to correctly answer three questions about interest rates, inflation, and investment risks	1.06	0.92	0.00	3.00
Concern	Never concerned (1), Very concerned (5)	2.22	1.09	1.00	5.00
Health	Healthy (1), Unhealthy (0)	0.87	0.34	0.00	1.00
**Household characteristic variables**					
Hpop	Total population of a household	3.40	1.54	1.00	20.00
Old	Percentage of population aged 60 and over in total	0.25	0.36	0.00	1.00
Pincome	Log household income per capita	9.58	1.31	−0.69	15.43
Wealth	Log wealth	12.83	1.48	3.60	16.81
House_if	Yes (1), no (0)	0.93	0.25	0.00	1.00
Social_exp	Log expenses for vocation, wedding, and funeral	7.84	1.14	0.00	10.31
Trust	Very distrustful (1), Very trusting (5)	2.20	0.90	1.00	5.00
**Regional characteristic variables**					
Pgdp	The GDP per capita of the province (unit: ten thousand yuan)	5.13	1.99	2.14	10.12

### Model Setting

#### Benchmark Regression Model

We choose the Probit model to estimate the impact of whether the household participates in medical insurance on the probability of household participation in the stock market. The Probit model is set as follows:

(1) $$\begin{array}{l} Stock\text{\_}if_{i}^{*}\;=\;\alpha_{1}Medical\text{\_}if_{i}\;+\;\beta_{1}X_{i}\;+\;\varepsilon_{i},\text{ where }Stock\text{\_}if_{i}\\ \;=\;I\left(Stock\text{\_}if_{i}^{*}\;>\;0\right) \end{array}$$

where *Stock_if*_*i*_ is a dummy variable set to one if household *i* participates in stock market. *Stock_if*_i_^*^ is a latent variable. *I*(·) is a symbolic variable; if the expression in parentheses is established, the value is 1; otherwise, it is 0. *Medical_if*_*i*_ is a dummy variable of medical insurance in household. *X*_*i*_ is a series of control variables, including the three types of characteristic variables. ε_*i*_ is the independent and identically distributed random disturbance item.

To estimate the effect of household participation in medical insurance on the proportion of equity asset, we choose the Tobit model, which is set as follows:

(2) $$\begin{array}{l} Stock\text{\_}rate_{i}\;=\;\max\left\{0,Stock\text{\_}rate_{i}^{*}\right\},\;where\;Stock\text{\_}rate_{\text{i}}^{*}\\ \;=\;\gamma_{1}Medical\text{\_}if_{i}\;+\;\delta_{1}X_{i}\;+\;\varepsilon_{i} \end{array}$$

where ${Stock\_rate}_{i}^{*}$ is a latent variable, and *Stock_rate*_*i*_ is the proportion of equity asset. Other variables have the same meanings as those in Equation (1).

#### Intermediary Effect Test Model

We refer to the mediating effect test model proposed by Baron and Kenny (34) and specify the following econometric model:

(3) $$\begin{array}{l} Stock\text{\_}if_{i},\;Stock\text{\_}rate_{i}=\;\theta_{i}\;+\;cMedical\text{\_}ifi+\eta_{1}X_{i}\;+\;\varepsilon_{i} \end{array}$$

(4) $$\begin{array}{l} {\text{Presave}}_{i}\;=\;\theta_{i}+aMedical\text{\_}ifi+\;\eta_{2}X_{i}\;+\;\varepsilon_{i} \end{array}$$

(5) $$\begin{array}{l} Stock\text{\_}if_{i},\;Stock\text{\_}rate_{i}\;=\;\theta_{i}\;+\;cMedical\;+\;bPresave_{i}\\ \;+\;\eta_{3}X_{i}\;+\;\varepsilon_{i} \end{array}$$

In Equations (3–5), other variables have the same meanings as those in Equation (1) above, *Presave*_*i*_ is the mediating variable, while *a, b, c*, and *c*′ are the regression coefficients. When the coefficients *a, b*, and *c* in the model are significant, there is a mediating effect. Besides that, if *c*′ is not significant, it is a complete mediating effect; otherwise, it is a partial mediating effect.

## Empirical Results and Analysis

### Benchmark Regression Results

We perform a series of empirical analysis of the relationship between whether the household participates in medical insurance and their participation in the stock market. Benchmark empirical results are shown in Table 4. The explained variable in regressions (1–3) is whether to hold stocks, and that in regressions (4–6) is the proportion of equity asset. Among them, regressions (1) and (4) are the estimation results under the full sample. We find that the regression coefficients of *Medical_if* are significantly positive, which means medical insurance plays a significant role in promoting stock market participation and the proportion of stock in financial assets. Households with medical insurance have lower financial risks caused by sudden illnesses and thus make a riskier decision than those without. In addition, we also subdivide medical insurance into social medical insurance (regressions 2 and 5) and other medical insurances (regressions 3 and 6)^6^. As mentioned before, the regression coefficients of the core explanatory variables are still significantly positive. The above regression results verify the hypothesis 1 of this paper.

**Table 4:** Benchmark regression results.

**Variables**	***Stock_if*** **(*****Probit*****)**			***Stock_rate*** **(*****Tobit*****)**		
	**(1)**	**(2)**	**(3)**	**(4)**	**(5)**	**(6)**
Medical_if	0.1673* (1.79)	0.1512** (2.27)	0.1490** (2.30)	9.8663** (2.28)	7.5215** (2.47)	4.6413* (1.65)
Male	−0.1769*** (−4.21)	−0.1790*** (−4.26)	−0.1727*** (−4.11)	−7.7505*** (−4.00)	−7.8250*** (−4.04)	−7.5968*** (−3.92)
Age	0.0711*** (6.34)	0.0702*** (6.26)	0.0713*** (6.38)	3.3391*** (6.42)	3.3050*** (6.34)	3.3811*** (6.50)
Age^2^/100	−0.0713*** (−6.00)	−0.0701*** (−5.88)	−0.0713*** (−5.99)	−3.3188*** (−5.98)	−3.2646*** (−5.87)	−3.3459*** (−6.03)
Married	0.1560** (2.20)	0.1533** (2.17)	0.1616** (2.28)	7.1290** (2.16)	7.0047** (2.13)	7.3952** (2.24)
Edu	0.0912*** (12.30)	0.0927*** (12.46)	0.0909*** (12.26)	4.0934*** (12.10)	4.1788*** (12.31)	4.1058*** (12.11)
Attitude	0.3553*** (12.51)	0.3556*** (12.52)	0.3534*** (12.46)	16.1860*** (12.19)	16.1921*** (12.20)	16.1091*** (12.13)
Know	0.0741*** (3.09)	0.0746*** (3.11)	0.0742*** (3.10)	2.9544*** (2.73)	2.9662*** (2.74)	2.9942*** (2.76)
Concern	0.2677*** (13.68)	0.2668*** (13.61)	0.2671*** (13.64)	12.4323*** (13.38)	12.3640*** (13.29)	12.4164*** (13.34)
Health_if	0.0280 (0.37)	0.0267 (0.35)	0.0230 (0.30)	1.9494 (0.55)	1.8601 (0.53)	1.6920 (0.48)
Hpop	−0.0302* (−1.77)	−0.0298* (−1.75)	−0.0295* (−1.73)	−2.2022*** (−2.71)	−2.1902*** (−2.70)	−2.2005*** (−2.70)
Old	0.1908** (2.05)	0.1937** (2.09)	0.1888** (2.03)	6.2539 (1.47)	6.4206 (1.51)	6.2377 (1.46)
Pincome	0.1162*** (5.22)	0.1161*** (5.22)	0.1157*** (5.22)	4.4236*** (4.42)	4.4240*** (4.42)	4.4115*** (4.42)
Nasset	0.3332*** (15.61)	0.3351*** (15.66)	0.3318*** (15.52)	14.7091*** (14.66)	14.7800*** (14.70)	14.6598*** (14.58)
House_if	−0.6339*** (−6.71)	−0.6389*** (−6.75)	−0.6275*** (−6.75)	−29.2243*** (−6.49)	−29.3619*** (−6.51)	−28.9518*** (−6.43)
Social_exp	0.0037 (0.19)	0.0042 (0.22)	0.0024 (0.13)	0.2652 (0.30)	0.2939 (0.33)	0.2352 (0.26)
Trust	−0.0075 (−0.30)	−0.0066 (−0.26)	−0.0088 (−0.35)	−0.6911 (−0.59)	−0.6620 (−0.57)	−0.7344 (−0.63)
Pgdp	0.0387*** (3.80)	0.0391*** (3.84)	0.0382*** (3.75)	1.5715*** (3.35)	1.5848*** (3.38)	1.5484*** (3.30)
N	11,863	11,863	11,863	11,863	11,863	11,863
Pseudo R^2^	0.3597	0.3600	0.3600	0.1488	0.1489	0.1487

***, **, and *, respectively, indicate significance at the 1, 5, and 10% level. The z-statistic value is in parentheses of the Probit model and the t-statistic value is in parentheses of the Tobit model.

The following results of control variables are from models (1) to (4). The coefficient of age (*Age*) is significantly positive, while the square coefficient of age (*Age*^2^/100) is significantly negative, which means age has an “inverted U” relationship with the household stock market participation probability, and so does the proportion of the stock market value. In other words, there is a significant life cycle effect in household stock market participation, which is the same as Fagereng et al. (35). Besides, the regression coefficients of marriage (*Married*), education (Edu), risk preference (*Attitude*), knowledge (*Know*), and financial attention (*Concern*) are all significantly positive. The wealth of married households is generally higher than that of unmarried households, so married households are more likely to participate in the stock market and hold a higher proportion of financial asset in stock market. The risk-preferred household is more adventurous and invests more assets in the stock market. Higher education level and financial attention enable people to obtain timely financial market information, which results in higher possibility of household participation in the stock market and larger proportion of equity asset. In addition, financial knowledge or literacy can reduce information search and adjustment costs on a complex asset such as stocks, which leads to a better household financial investment decision (36).

Furthermore, households with more members usually have heavier economic burden, which is not conducive to their participation in stock market. Therefore, the regression coefficient of the total population (*Hpop*) is significantly negative. In addition, the regression coefficients of household income per capita (*Pincome*) and wealth (*Wealth*) are significantly positive, indicating that richer households are more likely to participate in the stock market and increase their financial investment proportion because of lower per unit costs of stock market participation and adjustment (37). Finally, the regression coefficient of the GDP per capita of province (*Pgdp*) is significantly positive. It suggests that the level of regional economic development is an important factor affecting household stock market participation. Namely, the higher the level of economic development, the larger the possibility of household participation in the stock market, and proportion of equity asset.

### Endogenous Treatment

We use PSM to deal with the endogeneity in model estimation. The first step is to calculate the propensity score value and build a regression model with a binary dummy variable as the dependent variable. In the binary dummy variable, 1 represents the treatment group (households participating in medical insurance) and 0 represents the control group (households without medical insurance). Independent variables are the control variables of this paper. The formula for calculating the propensity score is: *P* = *Pr{DZ*_*i*_ =*1}* = ϕ*{X*_*i*_*}*, where *X*_*i*_ represents the control variables. The second step is to use an appropriate matching method to select the samples that are closest to the propensity score of treatment group samples as the final control group. In this paper, we use three matching methods: 1:1 nearest neighbor matching, kernel matching and radius matching. The estimation results of above methods are shown in Table 5, where ATT stands for average treatment effect on the treated. The results of ATT show that the net treatment effects are all significantly positive, further verifying the previous benchmark regression results.

**Table 5:** Endogenous test results.

**Matching method**	**(7)** ***Stock_if***			**(8)** ***Stock_rate***		
	**Treatment group**	**Control group**	**ATT**	**Treatment group**	**Control group**	**ATT**
Before	0.0942	0.0666	0.0276** (2.46)	3.0676	1.7828	1.2848*** (2.79)
1:1 nearest neighbor	0.0942	0.0684	0.0145* (1.78)	3.0673	1.6021	1.4652*** (2.92)
Kernel	0.0942	0.0698	0.0244** (2.33)	3.0673	1.9004	1.1669*** (3.21)
Radius	0.0942	0.0712	0.0230** (2.06)	3.0673	1.9147	1.1526*** (2.98)

***, **, and *, respectively, indicate significance at the 1, 5, and 10% level. The t-statistic value is in parentheses.

### Robustness Test

We have pursued a variety of robustness checks using the following methods. The OLS estimation results of regressions (9) and (10) in Table 6 show that participating in medical insurance can significantly increase the probability of participation in the stock market and the proportion of equity asset. When we replace the measure of explained variable with whether household holds risky financial assets (*Risk_if*), the proportion of risky financial assets (*Risk_rate*), and the types of risky financial assets (*Risk_number*)^7^. The results in regressions (11–13) show that participating in medical insurance can significantly increase the investment probability of risky financial assets, the proportion of risky financial assets, and the types of risky financial assets. Besides, considering that the samples of households with householders engaging in the financial industry may bias the estimation results of total samples, we make a re-estimation after removing these samples. The results in regressions (14) and (15) show that the coefficients of whether to participate in medical insurance (*Medical_if*) are still significantly positive. The tests of the above three methods prove that the previous benchmark regression results have a certain degree of robustness.

**Table 6:** Robustness test results.

**Variables**	**(9)**	**(10)**	**(11)**	**(12)**	**(13)**	**(14)**	**(15)**
	***Stock_if* (*OLS*)**	***Stock_rate* (*OLS*)**	***Risk_if* (*Probit*)**	***Risk_rate* (*Tobit*)**	***Risk_number* (*Oprobit*)**	***Stock_if* (*Probit*)**	***Stock_rate* (*Tobit*)**
*Medical_if*	0.0206** (2.32)	1.0091*** (3.07)	0.2350*** (3.21)	0.1218*** (3.46)	0.2366*** (3.56)	0.3437** (2.05)	18.2562*** (2.65)
Control	Yes	Yes	Yes	Yes	Yes	Yes	Yes
*N*	11,863	11,863	11,863	11,863	11,863	3,733	3,733
Pseudo *R*^2^	0.2574	0.1449	0.3426	0.3321	0.2838	0.3345	0.1312

*** and ** indicate significance at the 1 and 5% level respectively. The z-statistic value is in parentheses of the Probit model and the Oprobit model, and the t-statistic value is in parentheses of the OLS model and the Tobit model. The control variables here are the same as the previous benchmark regressions. Due to space limitations, the results of the control variables are not listed.

### Analysis of Heterogeneity

From the regression results of the control variables in the previous benchmark regression, household income per capita can significantly promote household stock market participation. To further examine the heterogeneous impact of different income, we generate a dummy variable *High*, which is assigned as 1 if the household income per capita is higher than the median, and 0 if not. The reference group is the sample of the household income per capita lower than the median. Then, the interaction term between medical insurance and High (Medical_if^*^High) is added to the model for estimation. As shown in the regressions (16) and (17) in Table 7, the coefficients of the interaction term (Medical_if^*^High) are significantly positive, implying that medical insurance has a more significant positive effect on the stock market participation probability and the proportion of equity asset in households with higher income per capita.

**Table 7:** Results of heterogeneity analysis.

**Variables**	**(16)**	**(17)**	**(18)**	**(19)**	**(20)**	**(21)**
	***Stock_if* (*Probit*)**	***Stock_rate* (*Tobit*)**	***Stock_if* (*Probit*)**	***Stock_rate* (*Tobit*)**	***Stock_if* (*Probit*)**	***Stock_rate* (*Tobit*)**
*Medical_if*	0.0287 (0.27)	2.3521 (0.48)	−0.5140^***^ (−3.58)	−23.3172^***^ (−3.48)	0.0484 (0.49)	5.0145 (1.09)
*Medical_if × High*	0.1937^***^ (2.89)	10.4607^***^ (3.29)	–	–	–	–
*Medical_if × City*	–	–	0.7366^***^ (6.48)	35.7457^***^ (6.64)	–	–
*Medical_if × East*	–	–	–	–	0.1887^***^ (3.04)	7.4961^***^ (2.62)
Control	Yes	Yes	Yes	Yes	Yes	Yes
*N*	11,863	11,863	11,863	11,863	11,863	11,863
Pseudo *R*^2^	0.3608	0.1495	0.3681	0.1529	0.3609	0.1492

*** *indicates significance at the 1% level. The z-statistic value is in parentheses of the Probit model and the t-statistic value is in parentheses of the Tobit model. The control variables here are the same as the previous benchmark regressions. Due to space limitations, the results of the control variables are not listed*.

We also generate a dummy variable *City* to study the different impact of urban and rural household medical insurance participation on stock market participation. The *City* is assigned as 1 if households locate in cities and towns, and 0 if not. Then, we put the interaction term between medical insurance and city (Medical_if^*^City) to the model for estimation. The results in regressions (18) and (19) show that the coefficients of the interaction term (Medical_if^*^City) are significantly positive. It means that medical insurance has a more significant positive impact on the possibility of household participation in the stock market and the proportion of equity asset in urban households.

To explore the different impact of household medical insurance participation on household stock market participation in regions, we add the interaction term between medical insurance and east (Medical_if^*^East) to the model for estimation (where *East* is a dummy variable set to one if household is in the eastern region). The results in regressions (20) and (21) suggest that compared with the central and western regions, medical insurance in the eastern region has a more obvious positive role in promoting household stock market participation.

Above all, medical insurance plays a more significant role in promoting the possibility of household participation in the stock market and the proportion of equity asset among households with high income level, urban and eastern households. Because these households have a strong ability to resist higher risks and less cost of participating in the stock market. Thus, the hypothesis 3 of this paper has been verified.

### Test of Impact Mechanism

We use the mediating effect test method to estimate the preventive savings effect of medical insurance. A mediating variable *Presave*, which is measured by the proportion of the sum of household cash and current deposits in financial assets, is introduced in estimation models. The results are shown in Table 8. Regressions (22–24) test the mediating effect in the relationship between medical insurance and the possibility of household participation in the stock market. The regression (22) shows that medical insurance (*Medical_if*) has a significant positive impact on the possibility of household participation in the stock market. And medical insurance (*Medical_if*) has a significant negative impact on precautionary savings in regression (23). When we put medical insurance and precautionary savings into the models, the results in regression (24) show that the coefficient of precautionary savings (*Presave*) is significantly negative, while that of medical insurance (*Medical_if*) is not significantly positive. It can be concluded that precautionary savings has a completely mediating effect. Similarly, the results in regressions (25–27) also indicate that precautionary savings have a completely mediating effect in the relationship between medical insurance and the proportion of equity asset. It can be inferred that medical insurance helps households reduce medical costs and precautionary savings, thus increasing the probability of stock market participation and the proportion of equity asset. Therefore, the hypothesis 2 of this paper has been verified.

**Table 8:** Test of influence mechanism.

**Variables**	**(22)**	**(23)**	**(24)**	**(25)**	**(26)**	**(27)**
	***Stock_if* (*Probit*)**	***Presave* (*Tobit*)**	***Stock_if* (*Probit*)**	***Stock_rate* (*Tobit*)**	***Presave* (*Tobit*)**	***Stock_rate* (*Tobit*)**
*Medical_if*	0.1673* (1.79)	−0.0476*** (−4.00)	0.0280 (0.27)	9.8663** (2.28)	−0.0476*** (−4.00)	2.6660 (0.67)
*Presave*	–	–	−1.7673*** (−31.79)	–	–	−76.4638*** (−28.45)
Control	Yes	Yes	Yes	Yes	Yes	Yes
*N*	11,863	11,863	11,863	11,863	11,863	11,863
Pseudo *R*^2^	0.3597	0.2796	0.4732	0.1488	0.2796	0.2061

***, **, and *, respectively, indicate significance at the 1, 5, and 10% level. The z-statistic value is in parentheses of the Probit model and the t-statistic value is in parentheses of the Tobit model. The control variables here are the same as the previous benchmark regressions. Due to space limitations, the results of the control variables are not listed.

## Conclusions and Policy Recommendations

This paper uses the data from CHFS in 2015 to study the relationship between household participation in medical insurance and the possibility of household participation in stock market and the proportion of equity asset. The results show that household participation in medical insurance significantly promotes the possibility of household participation in the stock market and the proportion of equity asset, and mainly through the preventive savings effect. Besides, the regions, urban–rural areas, and income level are also important influence factors. The medical insurance plays a more significant role in promoting participation in the stock market in households from eastern region, urban and households with higher income level. Furthermore, there is a significant life cycle effect in household stock market participation, and education, risk preference, financial knowledge, financial attention, household income, and wealth also have a significant effect on household participation in the stock market.

The following policy recommendations can be drawn from the empirical results. First of all, while improving Chinese medical insurance system, we should appropriately improve the level of medical security, so as to effectively reduce the risk of huge medical expenses for households. Besides, the design of medical insurance system should take into account the fact of unbalanced economic development among regions and urban–rural areas in China. And the preferred policy should be appropriately inclined to backward areas and rural areas to improve their medical security level. Simultaneously, it is necessary to increase rural financial support and accelerate the pace of inclusive finance development. Furthermore, related financial institutions or governments need to appropriately provide more financial training to improve financial attention of residents and capacity of participating in the financial market. Finally, government should further improve the social security system, such as pension, employment, education, etc., to a greater extent to reduce the precautionary savings and optimize the allocation of household financial assets.

In the future, we can explore international research on medical insurance policies and their impact on stock market participation. We can also conduct a detailed classification and discussion on the impact of a certain type of medical insurance reform on stock market participation or financial market participation.

## Data Availability Statement

Publicly available datasets were analyzed in this study. This data can be found here: China Household Finance Survey (https://chfs.swufe.edu.cn).

## Author Contributions

GS conceived and designed the experiments. ML and T-TS performed the experiments. YM completed the post-modification and proofreading of the paper. All authors contributed to the paper and approved the submitted version.

## Conflict of Interest

The authors declare that the research was conducted in the absence of any commercial or financial relationships that could be construed as a potential conflict of interest.

## Publisher's Note

All claims expressed in this article are solely those of the authors and do not necessarily represent those of their affiliated organizations, or those of the publisher, the editors and the reviewers. Any product that may be evaluated in this article, or claim that may be made by its manufacturer, is not guaranteed or endorsed by the publisher.

## Footnotes

^1^Data come from “China Urban Household Wealth Health Report 2019.” Among them, the data on the proportion of financial assets of urban households in the United States are from 2016, while those of Japan are from 2017.

^2^Data come from “China Health Statistics Yearbook 2019” and “China Statistics Yearbook 2019”.

^3^In 1998, the Chinese government promulgated the “Decision on Establishing a Basic Medical Insurance System for Urban Workers” and began to establish a basic medical insurance system for urban employees throughout the country. In 2003, it launched a pilot project for a new rural cooperative medical insurance system. In 2007, it introduced a basic medical insurance system for urban residents.

^4^Data are from the National Bureau of Statistics: http://www.stats.gov.cn/

^5^The medical insurance in this paper includes medical insurance for urban employees, medical insurance for urban residents, new rural cooperative medical insurance, medical insurance for urban and rural residents, public medical insurance, commercial medical insurance purchased by enterprises, commercial medical insurance purchased by individuals, enterprise supplementary medical insurance, medical treatment for serious diseases, social mutual aid, and other medical insurance. As long as the householder participates in one of the above medical insurances, the household is considered to have participated in medical insurance.

^6^The social medical insurance includes medical insurance for urban employees, medical insurance for urban residents, new rural cooperative medical insurance, and medical insurance for urban and rural residents, while others mainly include commercial medical insurance purchased by enterprises, commercial medical insurance purchased by individuals, enterprise supplementary medical insurance, and medical treatment for serious diseases.

^7^Since the types of risky financial assets held is a discrete choice in order, we choose the ordered Probit model (Oprobit) to estimate the relationship between medical insurance and the types of risky assets.
